# Advancements in the Research and Application of Whole-Plant Maize Silage for Feeding Purposes

**DOI:** 10.3390/ani15131922

**Published:** 2025-06-29

**Authors:** Xuelei Zhang, Xiaoxiao Liang, Yong Zhang

**Affiliations:** School of Biological Engineering, Henan University of Technology, Zhengzhou 450001, China; zhangxuelei1020@126.com (X.Z.); lxx2024@haut.edu.cn (X.L.)

**Keywords:** forage whole-plant maize silage, nutritional value, fermentation technology, sustainable development, planting techniques

## Abstract

This paper offers an exhaustive review of various pivotal aspects of forage whole-plant maize silage. It commences with an exploration of the foundational elements of planting, including the growing environment, variety selection, planting techniques, management practices, and harvesting considerations. The paper assesses the nutritional value of maize silage, its effects on animal health, and its current applications in livestock farming. Additionally, it elucidates the principles of fermentation, pathogen control, and the impact of fermentation technology on silage quality. The paper also discusses utilization strategies and technological advancements. A historical perspective is provided, alongside an analysis of current challenges, opportunities, and the global market positioning of maize silage. Furthermore, the paper delves into future prospects by addressing sustainable development strategies, adaptation to climate change, and ethical and economic controversies. The primary aim is to serve as a comprehensive reference for further research, production practices, and industrial chain development in the domain of forage whole-plant maize silage.

## Simple Summary

This paper presents an exhaustive review of forage whole-plant maize silage, encompassing its cultivation practices, nutritional advantages for livestock, and technological advancements in fermentation and storage. Although maize silage is extensively utilized in animal agriculture due to its efficiency and nutritional benefits, challenges remain in optimizing quality, managing pathogens, and adapting production processes to climate change. The study underscores the intersection of historical practices and contemporary innovations while identifying gaps in addressing sustainability, ethical considerations, and economic trade-offs. Despite widespread acknowledgment of its significance, the practical implementation of advanced techniques is inconsistent, particularly in balancing productivity with environmental and animal welfare priorities. By synthesizing global research and industry trends, this review seeks to guide future efforts in enhancing silage production, utilization, and sustainable development within the agricultural sector.

## 1. Basis of Cultivation of Forage Whole-Plant Maize Silage

### 1.1. Growing Environment and Variety Selection of Maize Silage

The growing environment plays a crucial role in determining the yield and quality of maize silage. Key environmental factors include soil temperature and growing degree days. Research indicates that the optimal soil temperatures for planting early-, mid-, and late-maturing maize varieties were at 16.6, 16.2, and 15.6 °C, respectively. Correspondingly, the optimal growing degree days at harvest for these maturity groups were at 1424, 1363, and 1542 °C [1]. Deviations from these optimal conditions led to a quadratic decline in biomass, with late-maturing maize varieties being particularly sensitive to fluctuations in temperature and growing degree days [1]. Different maize silage varieties exhibit varying performances under diverse climatic conditions. In cool climate cropping systems, the DKC26-28RIB and Yukon-R varieties demonstrate significantly greater leaf area, plant height, and biomass production compared to other genotypes. Biomass production is strongly and positively correlated with phosphatidate levels, suggesting its potential as a biomarker for selecting maize genotypes that are well-suited for silage production in cool climates [2]. In breeding programs, meiotic stability varies among commercial hybrids. For instance, hybrid SG6010 demonstrated the lowest average frequency of abnormal cells at 21.27%, whereas P30K64 exhibited the highest at 44.43%. Based on meiotic stability, hybrids such as CD304, P30K64, SG6010, and P30F53 are promising candidates for the development of new hybrids [3].

Furthermore, the effectiveness of maize silage varieties in livestock farming applications is influenced by their fermentation and nutritional parameters, which differ among hybrid types. Dual-purpose maize silage generally presents a higher lactic acid to acetic acid ratio, as well as increased levels of propionic acid, ether extract, and starch content, whereas silage-specific maize silage is characterized by a higher dry matter yield and neutral detergent fiber content [4]. The selection of whole-plant silage corn requires comprehensive consideration of variety characteristics, yield potential, nutritional quality, stress resistance, and adaptability. Priority should be given to those with high yield and high efficiency, strong stress resistance, and mechanical harvesting characteristics. At the same time, attention should be paid to green retention and the harvest period. BMR varieties have important application value in the field of silage corn, with reduced lignin content, high feed digestibility, and nutritional value.

### 1.2. Planting Technology and Management of Whole-Plant Maize Silage

**Figure 1:**
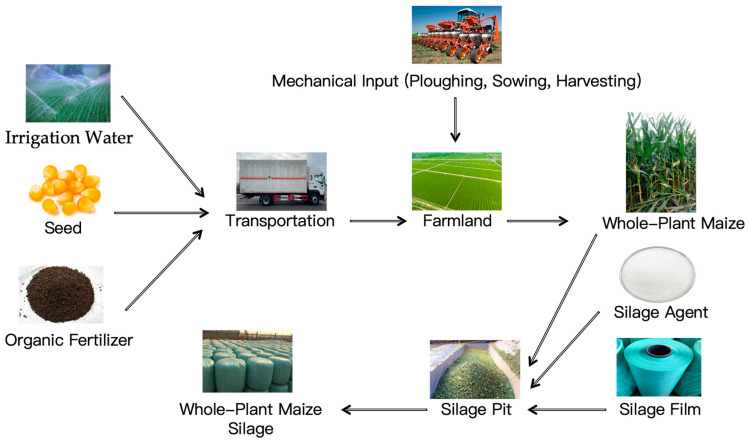
The cultivation and fermentation process for corn whole-plant silage.

The technology and management practices associated with the cultivation of whole-plant maize silage are crucial for attaining optimal yield and quality. The whole-plant silage corn planting and ensiling process is shown in Figure 1. In terms of planting density, empirical studies have indicated that the highest yields are achieved at a density of 79,500 plants per hectare across various maize varieties. For example, the Xunqing 858 variety demonstrated yield increases of 11.5% to 51.6% compared to three other varieties at this density. Additionally, this variety exhibited a plant height that was 8.7% to 22.6% greater than other varieties and a leaf area that was 30.9% larger than that of Xuntian 3171 [5]. Appropriate planting density can significantly improve the yield and resource use efficiency of corn. Under semi-arid climatic conditions, suitable planting density can improve the resource use efficiency and yield stability of rain-fed corn [6]. Increasing planting density can improve the photosynthetic capacity and biomass accumulation of corn, thereby achieving higher yields under high-density conditions [7]. However, too high planting density may lead to decreased photosynthetic capacity and unstable yield, so in semi-arid areas, moderate planting density is the key to achieving sustainable agricultural development [6]. Appropriate planting density combined with reduced nitrogen fertilizer application can achieve high yields while improving nitrogen fertilizer use efficiency and reducing environmental impact [8]. In studies in the Huanghuaihai region, appropriate planting density and nitrogen fertilizer application combinations can improve the profitability and nitrogen use efficiency of summer corn [9]. In addition, corn planting density is closely related to the variety’s lodging resistance. Modern corn varieties show stronger lodging resistance under high-density conditions, which is related to vertical changes in stem dry matter and nitrogen distribution [10]. By selecting varieties with smaller leaf angles, corn yield and lodging resistance can be improved under high-density conditions [11].

Fertilization management is also a critical factor, as optimizing nitrogen fertilizer application can significantly improve both the yield and quality of maize silage, particularly in cold northern regions. For instance, a comprehensive management strategy involving the Xunqing 858 variety, a planting density of 79,500 plants per hectare, and a polymer-coated urea-to-urea nitrogen ratio of 7:3 resulted in the highest yield of 73.1 t/ha. This strategy also produced silage with 11.1% crude protein, 19.1% starch, and the lowest neutral detergent fiber content of 50.6%. This approach significantly enhanced economic efficiency by 10.3% to 97.8%, improved nitrogen fertilizer use efficiency, reduced soil nitrate leaching, and minimized nitrogen surplus [5]. Reasonable nitrogen fertilizer management can improve nitrogen fertilizer use efficiency and reduce nitrogen loss. In arid areas, the mixed application of urea and slow-release nitrogen fertilizer can significantly improve the dry matter accumulation, nitrogen absorption, and nitrogen use efficiency of corn while reducing ammonia volatilization and soil nitrate nitrogen residue [12]. In another study, the application of urea–ammonium nitrate solution (UAN) can improve the dry matter accumulation and nitrogen use efficiency of corn and reduce N_2_O emissions compared with ordinary urea [13]. In semi-arid areas, the application strategy of replacing part of chemical nitrogen fertilizer with organic fertilizer has also been shown to maintain corn yield and improve nitrogen use efficiency. Replacing 30% of chemical nitrogen fertilizer with organic fertilizer can improve the dry matter accumulation and nitrogen absorption efficiency of corn while reducing the risk of environmental pollution [14]. In addition, deep nitrogen fertilizer application can improve the photosynthetic performance of corn after silking, thereby increasing corn yield [15]. In addition, the application of bio-organic fertilizer can improve corn yield and soil multifunctionality by regulating soil physical and chemical properties and microbial characteristics [16].

Furthermore, cutting height is a critical factor influencing the nutrient composition of maize silage. Specifically, a higher cutting height (e.g., 65 cm) in silage corn results in increased concentrations of dry matter and starch, decreased levels of neutral detergent fiber and lignin, and improved digestibility of neutral detergent fiber [17]. This change may be due to the fact that higher cutting heights reduce the fiber portion of the plant and increase the proportion of grains, thereby improving the overall nutritional value [18]. Higher cutting heights can improve the digestibility of silage corn. Studies have found that silage corn cut at higher cutting heights has improved neutral detergent fiber digestibility both in vivo and in vitro, which may be due to reduced fiber content and increased starch content [19]. This improvement in digestibility has a positive effect on the production performance of dairy cows because higher starch content and lower fiber content can increase dry matter intake and milk production in dairy cows [20]. In practical applications, choosing the right cutting height can optimize the nutritional content of silage corn, thereby improving dairy cow production efficiency and milk quality. Studies have shown that higher cutting heights can not only improve the nutritional value of silage corn but also reduce the loss of nutrients during the ensilage process [21]. Therefore, in the production of silage corn, a reasonable adjustment of cutting height is an important strategy to improve feed quality and dairy cow production performance [22].

Additionally, microbial inoculation plays an important role in the fermentation process of silage, and inoculation with different lactic acid bacteria can significantly affect the fermentation characteristics of silage. Inoculation with a microbial preparation containing Lactobacillus lactis and Lactobacillus buchneri can increase the acetic acid concentration during long-term storage and promote the production of lactic acid and acetic acid, thereby enhancing its antioxidant stability [17,23]. In addition, the combination of inoculation with Lactobacillus buchneri and Lactobacillus hilgardii has also been shown to improve the fermentation efficiency and stability of silage [24]. In another study, the combination of inoculation with Lactobacillus buchneri and Lactococcus lactis was found to effectively reduce the lactic acid to acetic acid ratio in silage while reducing the number of yeasts, thereby improving the antioxidant stability of silage [25]. In addition, the combination of inoculation with Lactobacillus buchneri and other lactic acid bacteria can also inhibit the growth of undesirable microorganisms, such as Clostridium and Enterobacter, thereby improving the fermentation quality of silage [26]. Inoculation with a microbial preparation containing a variety of lactic acid bacteria can improve the fermentation quality of silage by promoting the production of lactic acid and acetic acid. For example, inoculation with a microbial preparation containing Lactobacillus plantarum and Lactobacillus buchneri significantly increased the lactic acid and acetic acid content of silage while reducing pH and ammonia nitrogen content [27]. These research results show that microbial inoculation has great potential in improving the fermentation quality and stability of silage [28].

By employing scientific planting techniques and management practices, the overall benefits of whole-plant maize silage can be substantially improved.

### 1.3. Timing and Method of Harvesting Maize Silage

The timing and methodology of maize harvesting for silage play a crucial role in determining its nutritional value and its subsequent utilization in livestock production. Concerning the timing of harvest, empirical studies have indicated that methane emissions from cows diminish as the maturity of whole-plant maize at the time of harvest increases. For example, whole-plant maize harvested at various stages of maturity, very early (25% dry matter (DM), CS25), early (28% DM, CS28), mid (32% DM, CS32), and late (40% DM, CS40), demonstrated a linear reduction in methane production per unit of dry matter intake, decreasing from 23.0 g/kg (CS28) to 20.1 g/kg (CS40). Similarly, methane production relative to total energy intake also exhibited a linear decline, from 0.067 MJ/MJ (CS28) to 0.060 MJ/MJ (CS40) [29]. The methods of harvesting should not be underestimated, as optimizing factors such as harvest maturity, kernel processing, theoretical cutting length, and cutting height can preserve or enhance the nutritional quality of maize silage and subsequently improve milk yield in cows. For instance, cutting height has a significant impact on the nutrient composition and fermentation characteristics of maize silage. Elevated cutting heights are associated with increased dry matter and starch content, reduced neutral detergent fiber and lignin content, and enhanced digestibility of neutral detergent fiber [17]. Furthermore, they postpone the harvest affects the fermentation quality of maize stover silage. In southern regions, delaying the production of maize stover silage by 7 to 15 days post-harvest results in increased pH and acetic acid content, while lactic acid content decreases [30]. Therefore, selecting appropriate harvest timings and methods based on the intended use of maize silage and its impact on animal performance is crucial for maximizing benefits.

## 2. Nutritional Value of Whole-Plant Maize Silage and Its Impact on Animal Health

Maize silage is rich in nutrients, and its composition can vary due to several factors. The type of hybrid significantly influences nutritional parameters, with dual-purpose maize silage exhibiting higher levels of the lactic acid to the acetic acid ratio, propionic acid, ether extract, and starch compared to silage-specific maize silage [4]. The cutting height significantly influenced the nutrient composition of maize silage. Specifically, silage harvested at elevated cutting heights (65 cm) demonstrated increased concentrations of dry matter and starch, alongside reduced levels of neutral detergent fiber (NDF) and lignin, and exhibited enhanced NDF digestibility [17].

Furthermore, silage additives can be divided into six categories: homofermentative lactic acid bacteria, obligate heterofermentative lactic acid bacteria (hetLAB), combined inoculants, other inoculants, chemical additives, and enzyme preparations. The application of additives such as homofermentative lactic acid bacteria (homLAB), hetLAB, molasses, and their combinations (MIX) significantly improved the nutritional and fermentation characteristics of maize silage. These enhancements included increased dry matter digestibility (in vitro), elevated lactic acid bacteria counts, improved dry matter recoveries, and enhanced aerobic stability while concurrently reducing yeast and mold counts [31]. Notably, maize silage inoculated with homLAB exhibited the highest levels of lactic acid and soluble carbohydrates, coupled with the lowest levels of acetic acid, NH-N, and pH [32]. In some studies, the combined use of homLAB, hetLAB, and molasses (MIX) has been shown to improve the nutritional value and fermentation quality of silage at the same time. MIX treatment significantly increased the content of lactic acid and soluble sugars while reducing the content of acetic acid and ammonia nitrogen [32]. In addition, the application of lactic acid bacteria can also improve the quality of silage by regulating the microbial community. The application of lactic acid bacteria can increase the relative abundance of lactic acid bacteria and reduce the presence of unfavorable bacteria, thereby improving the fermentation quality and stability of silage [33]. In some cases, the application of lactic acid bacteria can further improve the quality of silage by producing metabolites with antimicrobial activity [34]. In conclusion, the application of additives such as homo- and heterofermentative lactic acid bacteria, molasses, and their combination can significantly improve the nutritional and fermentation characteristics of corn silage in multiple ways. These additives not only improve the fermentation efficiency of silage but also enhance its stability under different environmental conditions.

Whole corn silage has a significant effect on improving animal health and production performance. It has a positive impact on animal health by improving digestive characteristics, increasing microbial diversity, and reducing greenhouse gas emissions. First, whole corn silage can improve rumen fermentation and the growth performance of ruminants. Studies have shown that feeding whole corn silage can improve the growth performance and rumen fermentation efficiency of cattle by changing the rumen microbiome [35]. Increasing the maturity of whole corn silage can effectively reduce methane emissions from dairy cows without affecting their production performance [29]. In the context of beef cattle farming, the incorporation of whole-plant maize silage has been shown to significantly enhance daily weight gain and decrease the feed-to-weight ratio (F/G) when compared to maize stover silage. Specifically, beef cattle consuming whole-plant maize silage exhibited increased daily weight gain and a reduced F/G ratio, which was associated with lower rumen acetic acid content and a decreased acetic acid to propionic acid ratio (A/P) [35]. In dairy cows, the stage of harvest maturity of whole-plant maize silage was pivotal, as it was observed that methane emissions declined with increasing maturity, without negatively impacting DM intake, milk yield, or milk composition. For instance, whole-plant maize harvested at varying maturity stages demonstrated a linear decrease in methane emissions per unit of fat- and protein-corrected milk volume [29]. For sheep, whole corn silage also has a positive effect. Studies have shown that whole corn silage treated with lignocellulose-degrading bacteria can improve the growth performance and rumen fermentation efficiency of sheep [36]. In addition, sunflower silage, which partially replaced whole corn silage, had no adverse effects on the growth performance and slaughter performance of Tan sheep, indicating that it can be used as an alternative feed resource to whole corn silage [37]. In pig breeding, whole corn silage also shows positive effects. Adding whole corn silage can improve and enhance the fiber digestion characteristics and intestinal microbial diversity of pigs [38]. In the study of ruminants, we can also refer to the research methods of Ran T et al. [39] and use omics technology to explore the effects of plants on body health. In another study, adding whole corn silage significantly improved the growth performance and serum biochemical indicators of pigs while increasing the diversity of intestinal microorganisms [40]. Research has demonstrated that pigs fed diets incorporating whole-plant maize silage from a body weight of 90 kg to a slaughter weight of 170 kg exhibited increased NDF content in their stomach contents, as well as greater gastric organ weight and pyloric region area. Additionally, there was a significantly lower incidence of follicular gastritis and a reduction in the severity of gastritis observed [41]. These findings suggest that the judicious use of whole-plant maize silage can positively influence the health and performance of various animal species. Compared with sorghum silage and alfalfa silage, corn silage has comprehensive advantages in nutritional value, yield, palatability, and planting adaptability. Although sorghum silage has excellent drought and salt tolerance, its nutritional value is slightly lower than that of corn. Although alfalfa silage has a high protein content, it needs to rely on additives to improve the silage effect and has a relatively low yield. Therefore, corn silage has become the first choice for ruminant feed with its comprehensive advantages.

Corn silage holds a pivotal role in animal husbandry. Globally, the increasing population in developing countries, coupled with rising meat consumption and the growing demand for animal feed, has driven the widespread adoption of corn silage. The global corn silage market is projected to expand at a compound annual growth rate of 7.84% from 2021 to 2030 [42]. In the northwestern plains of India, corn serves as a crucial alternative crop to rice, contributing to crop diversification due to the increasing scarcity of water resources, the reduction in farm diversity, nutrient depletion, and environmental pollution caused by rice straw burning. Owing to its rapid growth, high biomass yield, favorable palatability, and absence of anti-nutritional factors, whole-plant corn silage has become a prevalent high-energy, low-protein feed for dairy cows and buffaloes. It is frequently utilized in conjunction with high-protein feeds, such as alfalfa [42]. Research indicates that the incorporation of whole-plant corn silage can enhance rumen fermentation and cattle growth performance by altering the rumen microbial community. Specifically, whole-plant corn silage has been shown to significantly increase the daily weight gain of cattle and decrease the feed-to-body weight conversion ratio while also reducing acetic acid content and the acetic acid to propionic acid ratio in the rumen [43]. Furthermore, the partial substitution of high-fiber feed with silage corn has been associated with reduced methane emissions and improved feed conversion efficiency throughout the lactation cycle. Studies have demonstrated that increasing the proportion of silage corn in the diet can elevate dry matter intake and milk production while concurrently decreasing methane production and intensity [44]. In a separate study, the incorporation of silage corn containing α-amylase was demonstrated to enhance the lactation performance of dairy cows, augment milk protein and lactose production, and decrease methane emission intensity [45]. Regarding rumen fermentation, the combination of silage corn with various nitrogen source supplements has been shown to enhance the efficiency of microbial protein synthesis. Supplementation with urea, mixed amino acids, or protein improves the degradation of organic matter and crude protein in silage corn and increases fiber degradation capacity [46]. Furthermore, the integration of silage corn with linseed oil significantly reduces methane emissions without adversely affecting animal production performance [47]. Substituting grass silage with silage corn in dairy cow diets effectively reduces methane emissions while maintaining production performance. Research indicates that increasing the proportion of silage corn results in a linear reduction in the intake of neutral detergent fiber and crude protein while increasing starch intake, thereby reducing methane production [48].

## 3. Pathological Mechanisms and Fermentation Technology of Whole-Plant Maize Silage for Feeding Purposes

### 3.1. Fermentation Principles and Microbiological Action of Maize Silage

The fermentation process of corn silage is complex and involves the activity of multiple microorganisms. The basic principle of fermentation is based on the anaerobic conversion of carbohydrates into organic acids by microorganisms, thereby reducing the pH and preserving the feed. It has been observed that the combination of inoculation with Lactobacillus plantarum and Lactobacillus brucei (INOC1) can effectively reduce the proportion of lactic acid while increasing the proportion of acetic acid, thereby improving the fermentation quality and stability of silage [21,48,49]. Conversely, inoculation with Saccharomyces cerevisiae strain 3 (INOC2) has been found to enhance the aerobic stability of silage, reduce the rumen acetic acid concentration, and improve the apparent whole gut digestibility of dry matter and organic matter [50].

Furthermore, alterations in the microbial community dynamics significantly impact the fermentation process. During the fermentation of whole-plant maize silage, the initial aerobic phase is characterized by an increased abundance of Pantoea, Klebsiella, and other bacteria, which coincides with a rise in pH levels. As fermentation advances, the process transitions into an intense fermentation phase, marked by a rapid decline in pH and exponential microbial growth, with a shift in the dominant species from Pantoea to Weissella and subsequently to Lactobacillus. In the stabilization phase, both pH levels and microbial populations reach equilibrium, with Lactiplantibacter remaining the predominant species [51]. A comprehensive understanding of these fermentation principles and the roles of various microorganisms can facilitate the optimization of the silage fermentation process.

### 3.2. Control and Prevention of Pathogens in the Silage Process

The presence of pathogens during the silage process can adversely affect silage quality and animal health, necessitating effective control and prevention measures. Common pathogens include Enterobacter, Listeria, Bacillus, Clostridium, and Salmonella. For instance, Listeria can cause mortality and abortion in dairy cows, while Clostridium may result in mild diarrhea and reduced feed intake [52]. To mitigate the proliferation of these pathogens, the application of specific additives has proven effective. For example, the incorporation of pediocin SA-1, a bacteriocin synthesized by Pediococcus acidilactici, has been demonstrated to effectively inhibit the growth of Listeria monocytogenes in maize silage, thereby enhancing both fermentation quality and aerobic stability [53]. Similarly, the introduction of lactic acid bacteria or propionic acid has been documented to suppress the proliferation of *E. coli* O157:H7 in alfalfa silage. In instances where alfalfa silage was inoculated with *E. coli* O157:H7, the addition of lactic acid bacteria (specifically Lactobacillus plantarum or Lactobacillus buchneri) or propionic acid resulted in the pathogen becoming undetectable during the later stages of fermentation. Moreover, the inclusion of Lactobacilli facilitated a more rapid decline in pH and an increase in lactic or acetic acid concentrations [54].

Furthermore, the regulation of factors such as dry matter content and temperature is crucial in preventing pathogen proliferation. Environments characterized by elevated temperatures and humidity levels are particularly favorable for the growth of harmful microorganisms. Appropriately reducing dry matter content can also contribute to a decrease in the prevalence of certain antibiotic resistance genes. For instance, the prevalence of integrase and plasmid in whole maize silage is lower at a dry matter content of 30% compared to 40%. Moreover, inoculation with Lentilactobacillus buchneri can mitigate the abundance of high-risk antibiotic resistance genes by modulating pathogens and restricting the transfer of genetic elements that facilitate antibiotic resistance [55]. Improving the aerobic stability of corn silage after opening requires coordinated management of multiple links. First of all, in terms of material management, the vertical storage and access method is adopted to reduce air infiltration, keep it clean, and cover it with a sealing film. It is also necessary to apply additives scientifically. Acetic acid–propionic acid composite organic acid preparations can be sprayed before opening the warehouse. The quality monitoring system needs to establish a weekly inspection system for aflatoxin B1. The above technical combination can prolong the aerobic stability of corn silage, control the dry matter loss rate within 8%, and reduce the risk of mycotoxin contamination by more than 70%.

### 3.3. Effect of Fermentation Technology on Maize Silage Quality

**Figure 2:**
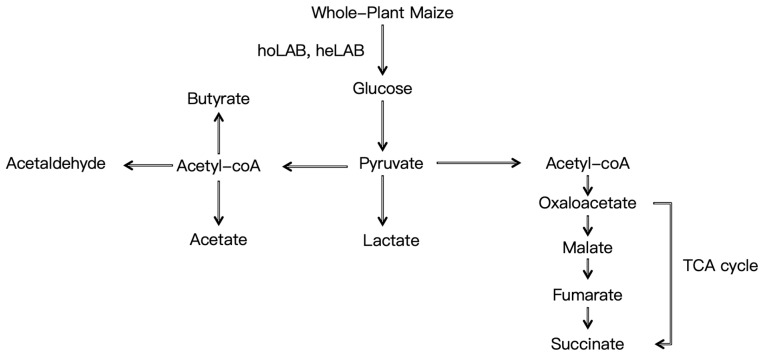
Diagram of the biochemical pathway of silage fermentation.

Fermentation technology plays a crucial role in determining the quality of maize silage. The use of additives and specific filling methods are pivotal factors in this process. Research indicates that the application of the Sila-Max™ (MAX) additive, in conjunction with a single filling technique, enhances the quality of whole-plant maize silage. This treatment results in the highest dry matter content, minimal dry matter loss, improved digestibility of acid detergent fiber (ADF), NDF, and crude protein (CP), as well as superior silage quality from primary filling compared to tertiary filling [56]. Additionally, inoculation with various bacterial strains influences both fermentation processes and silage quality. For instance, the inoculation of silage with bacterial agents containing Pediococcus pentosaceus and Propionibacterium freudenreichii (B2) led to an increased concentration of acetic acid. Conversely, the introduction of Lactobacillus buchneri 40,788 (BUC) resulted in a reduction in pH and propionic acid concentration, an increase in lactic acid concentration, and a decrease in DM loss (5.0% compared to 14.3%) [57]. Furthermore, the application of enzymes has been demonstrated to be effective in enhancing silage quality. The incorporation of cellulase and laccase improved the fermentation quality of mixed maize stover and wet brewers’ grains silage. Relative to the control, cellulase significantly elevated CP, water-soluble carbohydrates, and lactic acid bacteria counts while reducing the content of neutral detergent fiber and acid detergent fiber. Laccase significantly decreased acid detergent lignin content, and the combined application of both enzymes proved even more efficacious, further enhancing fermentation quality by increasing the abundance of Firmicutes and Lactobacillus spp. and reducing microbial diversity [58]. In summary, the optimization of fermentation technology can substantially improve the quality of maize silage. The biochemical pathway of silage fermentation is shown in Figure 2.

## 4. Strategies and Technological Progress in the Utilization of Whole-Plant Maize Silage for Feeding Purposes

### 4.1. Application of Maize Silage in Feed Formulation

In the context of feed formulation, maize silage is a prevalent component, known for its varied effects on animal performance. Research on beef cattle fattening has investigated the consequences of substituting corn grain with corn silage in diets that include corn-modified distillers, grains, and solubles (MDGS). It has been observed that dry matter intake (DMI), average daily gain (ADG), and the feed-to-weight gain ratio (G:F) decrease linearly as the proportion of corn silage increases from 15% to 55% on a dry matter basis. Specifically, beef cows consuming diets with 15% corn silage demonstrated G:F ratios that were 1.5%, 5.0%, and 7.7% higher than those of cows fed diets containing 30%, 45%, and 55% corn silage, respectively [59]. In dairy farming, the substitution of certain feed ingredients with whole-plant maize silage can affect cow performance. For example, in the spring, cows supplemented with maize silage, sugar beet silage, or high-moisture maize exhibited no significant differences in milk yield or fat concentration when sugar beet silage was used as a supplement compared to maize silage or high-moisture maize. The concentration of milk protein was observed to be lower in the group fed with sugar beet silage compared to those receiving high-moisture maize silage, as reported by Smith et al. [60]. Substituting certain dietary components with whole flint maize silage in calf diets has been shown to influence both intake and growth metrics. Empirical evidence indicates that incorporating 10% whole-plant flint maize silage into calf diets results in increased dry matter intake and average daily weight gain while also enhancing rumination and chewing time and reducing the risk of acidosis [61]. The strategic use of maize silage in feed formulations can fulfill the nutritional needs of various livestock species and enhance agricultural productivity.

### 4.2. Processing and Preservation Techniques for Maize Silage

The processing and preservation of maize silage are crucial for maintaining its quality. Factors such as chop length and mechanical handling play significant roles in determining utilization efficiency. For instance, maize silage harvested at the blackline maturity stage, with a theoretical cut length (TLC) of 19 mm and a roll gap of 3 mm or less, has been shown to exhibit higher apparent whole gut digestibility of starch. Optimizing the particle size of maize silage is essential for improving its utilization efficiency in dairy cows [62]. In the context of preservation technology, wet anaerobic storage has been identified as an effective method. Empirical studies indicate that traditional silage techniques result in a total solids dry matter loss (DML) of less than 6% for wet maize stover. This loss is approximately one-fifth of that observed in aerobic storage and slightly less than half of the loss associated with the anaerobic modified Ritter pile method. A field demonstration involving the storage of 272 dry tons of corn stover over a six-month period reported an average DML of less than 5% [63]. Furthermore, pretreatment strategies can enhance the efficiency of saccharification and enzymatic digestion of maize silage. For example, a two-step pretreatment process for maize stover silage, initially involving treatment with a non-ionic surfactant (Tween-80) at 60 °C for 60 min to remove 30.48% of lignin, followed by treatment with ferric nitrate at 130 °C for 30 min to remove 94.56% of hemicellulose, achieves a cellulose purity of 72.53% and a maximum enzymatic digestibility of 90.98%. This represents an increase of approximately 32.07% compared to the digestibility achieved with only the Tween-80 pretreatment [21]. Thus, the implementation of appropriate processing and preservation techniques is crucial for maintaining the quality and utilization value of maize silage.

### 4.3. Research and Application of Novel Silage Technologies

The advent of novel silage technologies presents innovative strategies to enhance the quality and utilization efficiency of maize silage. In the realm of whole-plant maize silage harvesting technology, optimizing parameters such as harvest maturity, kernel processing, theoretical cutting length, and cutting height is crucial for maintaining or enhancing the nutritional value of maize silage, thereby supporting dairy cow milk production. By precisely regulating these factors, the nutritional requirements of dairy cows can be more effectively met, leading to improved breeding efficiency [64]. In the domain of microbial inoculation technology, the selection of microorganisms with specific functional attributes for silage inoculation has emerged as a significant research focus. For example, Lactobacillus brevis 5M2 and L.buchneri 6M1, which exhibit anti-fungal and carboxylesterase activities, have been isolated from farm-scale maize silage. Their application as inoculants has been shown to enhance nutrient digestibility in the rumen, reduce yeast populations, and improve overall silage quality [65]. Furthermore, certain pretreatment technologies are being investigated for their potential synergistic effects when combined with the silage process. The pretreatment of maize silage using the white-rot fungus Trametes versicolor has been shown to enhance biogas production during anaerobic co-digestion with cattle manure. In a semi-continuous pilot-scale experiment, substituting a portion of the feedstock with pretreated maize silage resulted in an increase in the average methane production rate from 0.167 m^3^CH₄ kgVS^−1^ to 0.236 m^3^CH₄ kgVS^−1^ [66]. The exploration and implementation of innovative silage technologies are anticipated to propel further advancements within the maize silage industry.

## 5. Historical Evolution and Current Analysis of Whole-Plant Maize Silage for Feeding Purposes

### 5.1. Historical Development of Maize Silage Cultivation and Utilization

As a significant feed crop, maize silage has a long-standing and evolving history of cultivation and utilization. Initially, its cultivation was predominantly concentrated in countries with advanced animal husbandry sectors, such as the United States and various European nations. However, with the progression of agricultural technology and the growing demand for efficient feed sources, the cultivation of maize silage has progressively expanded to a broader range of regions. In developing countries, such as China and India, the population is growing rapidly, meat consumption is increasing, and the demand for animal feed is rising. Corn silage, due to its high energy and low protein properties, is an ideal feed choice for livestock, such as dairy cows, making it an important feed in agricultural production in these countries [42]. Recent investigations have demonstrated that distinct silage corn varieties exhibit considerable variation in nutritional value, as evidenced by analyses of their nutritional composition, rumen degradability, and total digestive tract digestibility [67]. Furthermore, empirical studies indicate that factors such as harvest timing, cutting length, and mechanical processing methods significantly influence the nutritional value of silage corn and its impact on dairy cow production performance [62]. With regard to the utilization of silage corn, contemporary research endeavors have concentrated on augmenting its nutritional value and utilization efficiency through advancements in planting and processing methodologies. For instance, optimizing parameters such as harvest maturity, grain processing, theoretical cutting length, and cutting height can enhance the fiber and starch digestibility of silage corn, thereby boosting milk production in dairy cows [68]. Furthermore, research indicates that integrating silage corn with other feed types, such as high-moisture corn or sugar beet silage, enhances the production performance of dairy cows, particularly under restricted grazing conditions [60]. On a global scale, the cultivation and utilization of silage corn are influenced by climate change and agricultural policies. In certain regions, effective water resource management and fertilizer use efficiency have emerged as critical factors impacting the yield and quality of silage corn [69]. Additionally, the cultivation of silage corn is intricately linked to alterations in soil fertility and microbial communities, collectively influencing the growth and nutritional content of the crop [68]. In summary, the cultivation and utilization of silage corn have advanced significantly over the past few decades. Ongoing research and technological advancements have positioned silage corn as an increasingly vital component in enhancing the production efficiency and sustainability of animal husbandry.

### 5.2. The Role of Maize Silage in the Global Market

Silage corn occupies an important position in the global market. In European and American countries, silage corn is an important source of feed for dairy cows, beef cattle, and other livestock. Its yield and quality directly affect the production efficiency of animal husbandry. In the United States, silage corn is widely used in dairy farming to provide essential nutrients for dairy cows, thereby ensuring the quantity and quality of milk production. In developing countries, such as China, population growth and improved living standards have led to increased consumption of meat and dairy products, which has stimulated the growth of the silage corn industry. Policy measures have promoted the optimization of the food–economy–feed structure and the expansion of feed crop planting, including silage corn. As a result, the yield and quality of silage corn have been significantly improved, meeting the demand for high-quality feed in the animal husbandry industry.

The nutritional value of silage corn is largely contingent upon its maturity at harvest and the conditions under which it is stored. The dry matter content at harvest significantly influences the starch content and the starch-to-neutral detergent fiber ratio in silage corn, which is directly associated with the dry matter intake, milk yield, and milk protein content in dairy cows [70]. Furthermore, the fermentation quality of silage corn is pivotal to its nutritional value. Research indicates that employing heat-resistant lactic acid bacteria during silage fermentation enhances fermentation outcomes under elevated temperature conditions, thereby augmenting the nutritional value of silage corn [71]. Variability in fermentation and nutritional parameters is also observed among different corn hybrids. Dual-purpose corn silage exhibits superior unit nutritional value, whereas dedicated silage corn excels in biomass yield [4]. In the context of dairy cattle breeding, incorporating silage corn can enhance the dry matter intake and milk production of dairy cows. Empirical evidence suggests that supplementing a grass silage-based diet with silage corn substantially increases the dry matter intake and milk yield of dairy cows [70]. Additionally, the high-fiber digestibility of silage corn supports increased dry matter intake and milk production [67]. The technology for harvesting and storing silage corn is continuously advancing to enhance its nutritional value and economic benefits. Technical measures, including the optimization of harvest maturity, grain processing, cutting length, and cutting height, have been shown to improve the nutritional quality of silage corn and enhance the milk production performance of dairy cows [64]. Furthermore, the application of dual-purpose lactic acid bacteria inoculants can enhance the fermentation quality of silage corn and minimize nutritional losses [72]. In conclusion, as a significant feed component in the global market, the nutritional value and economic advantages of silage corn remain subjects of ongoing research and interest. By refining harvesting and storage techniques, along with the selection of appropriate corn varieties and fermentation strains, the efficiency of utilization and market competitiveness of silage corn can be further enhanced.

Concerning market size, the global corn silage market is experiencing growth and is projected to expand at a compound annual growth rate of 7.84% from 2021 to 2030, driven by factors such as the development of animal husbandry and the rising demand for sustainable feed [42]. Simultaneously, silage corn presents a distinct price advantage within the feed market. When compared to other feed raw materials, its cost-effectiveness is more competitive in certain regions, thereby reinforcing its position in the global market.

### 5.3. Current Challenges and Opportunities in the Maize Silage Industry

The maize silage industry is presently confronted with a series of challenges and opportunities. Regarding challenges, climate change significantly impacts maize silage growth during the planting stage; alterations in temperature and precipitation patterns may necessitate adjustments in planting areas, schedules, and varietal adaptations. Additionally, pests and diseases continue to pose substantial threats, adversely affecting both yield and quality. During the silage process, there exists a considerable risk of contamination by pathogens, such as Enterobacteriaceae and Listeria monocytogenes, which can compromise silage quality and jeopardize animal health [51]. Furthermore, the market lacks adequately developed hybrids specifically designed for silage production, complicating efforts to satisfy diverse demands.

Nevertheless, the industry is also presented with numerous opportunities. The global increase in population and the rising demand for meat and dairy products have concurrently escalated the demand for animal feed, thereby propelling the expansion of the silage corn market. Projections indicate that the global corn silage market will experience a compound annual growth rate of 7.84% from 2021 to 2030 [42]. To adequately address this demand, it is imperative for producers to enhance yields without compromising the nutritional quality of silage corn. This necessitates optimization of various factors, including harvest maturity, grain processing, cutting length, and cutting height, to sustain or enhance the nutritional value and milk production of lactating dairy cows. Furthermore, advancements in technology have introduced novel tools for dairy farmers and corn growers aimed at improving the digestibility of fiber and starch in silage corn. Recent innovations in processing equipment have emerged, enhancing harvesting efficiency and optimizing crop processing parameters. Future research should focus on evaluating the impact of these new processing technologies and developing appropriate assessment methodologies to refine nutritional modeling and enhance production efficiency [64]. At the technical level, advancements in planting and silage technologies are continually emerging. These include optimized harvesting techniques designed to enhance the nutritional value of silage corn, as well as microbial inoculation technologies aimed at improving silage quality. Furthermore, with the increasing emphasis on sustainable agriculture, silage corn, recognized as a relatively environmentally friendly feed source, holds significant potential for development when integrated with sustainable cultivation and utilization practices.

## 6. Future Prospects and Points of Contention for Forage Whole-Plant Maize Silage

### 6.1. Sustainable Development Strategies for Maize Silage Cultivation

Implementing a sustainable development strategy for maize silage cultivation is imperative. In the realm of variety selection, it is crucial to intensify the breeding of specific hybrids tailored for silage production. This should focus on enhancing traits such as dry matter yield, nutrient yield, energy content, the genetic structure of cell wall components and their digestibility, stalk uprightness, maturity span, and minimizing silage loss. For instance, molecular breeding techniques can be utilized to identify genes associated with specific traits, facilitating the development of crop varieties that are better adapted to diverse environmental conditions and requirements [42]. Furthermore, microbial fungicides can establish symbiotic associations with maize seeds or root systems through seed dressing or basal fertilizer application, thereby promoting root development, increasing yields, enhancing resilience, and improving soil health, ultimately enhancing production efficiency. In the context of planting management, resource optimization is of paramount importance. Specifically, in the northern cold zone, integrated management strategies, such as the rational selection of varieties, appropriate planting density, and optimized nitrogen fertilizer management, can improve the yield and quality of maize silage. These strategies also enhance nitrogen fertilizer utilization rates, reduce soil nitrate leaching and nitrogen surplus, and align with the principles of sustainable agriculture [5].

Moreover, the promotion of sustainable irrigation and fertilizer application techniques, such as precision irrigation and formula-based fertilizer application, aims to enhance the efficiency of water and fertilizer use while minimizing resource wastage and environmental pollution. In terms of silage, the use of fermented maize silage and maize by-products supports long-term storage and improves farming efficiency. Effective waste utilization strategies can be employed to process and utilize corn stover and other agricultural residues, for instance, through mixed silage with other agricultural wastes or bio-energy production. These strategies facilitate resource recycling, alleviate environmental pressures, and foster the sustainable development of maize silage cultivation.

### 6.2. Adaptation Studies of Maize Silage in a Changing Climate

Adaptation studies of maize silage in the context of a changing climate are increasingly important, as climate change presents significant challenges to maize silage cultivation. Changes in temperature and precipitation patterns affect the growth, development, and yield quality of maize silage, underscoring the need for adaptation research. Research has indicated that maize silage genotypes exhibit differential responses to climatic conditions. Notably, the DKC26-28RIB and Yukon-R varieties demonstrate increased leaf area, plant height, and biomass production in cooler climates. Phosphorus accumulation (PA) has been identified as a potential biomarker for screening genotypes suited to cooler environments [2]. Adaptations in crop management practices are essential to address the challenges posed by climate change. For instance, sowing schedules can be adjusted based on climatic forecasts, with delayed sowing recommended in regions experiencing elevated temperatures to mitigate the adverse effects of high temperatures on maize growth. In drought-prone areas, the selection and promotion of drought- and heat-tolerant varieties are crucial to reducing the impact of water stress on yield. Furthermore, soil amendments, such as biochar, can improve soil structure and enhance water and nutrient retention, thereby increasing the adaptability of maize silage to climate change. Empirical studies have demonstrated that the application of biochar reduces greenhouse gas emissions, enhances soil fertility, and supports the maintenance of yield and quality in maize silage under varying climatic conditions [72].

### 6.3. Economic Controversies in the Utilization of Maize Silage

From an economic perspective, the cost-effectiveness of maize silage remains a contentious issue. The processes of cultivation, harvesting, and silage production entail various expenses, including those associated with seeds, fertilizers, machinery, equipment, and additives. Market price fluctuations and inefficiencies in farming practices can adversely affect the economic returns for producers. For instance, in certain regions, maize silage production is highly susceptible to climate variability, resulting in significant supply fluctuations. Given the relatively stable market demand, these variations can lead to price volatility, thereby impacting the income of growers. Furthermore, the economic returns associated with different maize silage varieties and utilization methods exhibit considerable variability. While some high-quality varieties provide high yields and superior quality, they also incur higher seed costs. Consequently, a comprehensive cost–benefit analysis is essential to ascertain the optimal planting and utilization strategy.

The cost–benefit analysis of corn silage includes direct costs and indirect costs. Among them, direct costs include seeds and planting inputs, fertilizers and pesticides, irrigation and labor, and mechanical operations, and indirect costs include land rent, equipment depreciation, and management expenses. Corn silage has the advantages of controllable costs, large yield potential, and stable market demand, and has significant economic benefits. By optimizing planting management, improving quality standards, and integrating industrial chain resources, the benefits can be further increased. It is a high-quality choice for the coordinated development of modern agriculture and animal husbandry.

### 6.4. The Environmental Benefits of Maize Silage Production

Corn silage production has important environmental benefits in agriculture, especially in terms of sustainable agriculture and environmental protection. First, corn silage production can reduce greenhouse gas emissions in a variety of ways. For example, studies have shown that the addition of biochar can significantly reduce greenhouse gas emissions from corn silage production, including emissions of carbon dioxide, methane, and nitrous oxide [72]. In addition, the application of biochar can also increase the carbon storage capacity of the soil, thereby further reducing the global warming potential [73]. Second, the covering materials used in the production of corn silage, such as corn cobs, can effectively reduce ammonia volatilization losses, thereby improving fertilizer utilization efficiency. This approach not only reduces ammonia emissions but also increases the fertilizer value of stored dairy wastewater [74]. In addition, the co-storage of corn silage with other crop residues, such as co-storage with oregano oil extraction residues, can improve fermentation quality and reduce the risk of proliferation of undesirable bacteria, thereby improving the quality of silage [75]. In terms of soil management, corn silage production can improve soil health through reasonable fertilizer management and soil improvement measures. For example, applying high-phosphate dairy manure can significantly improve soil biochemical properties and microbial community activity, thereby increasing crop phosphorus use efficiency and yield [76]. In addition, adopting a no-till system and retaining wheat residues can reduce the emission of environmental pollutants and is a more sustainable production method [77]. In summary, silage corn production has significant environmental benefits in reducing greenhouse gas emissions, improving fertilizer use efficiency, improving soil health, and reducing environmental pollution. These measures not only help achieve sustainable agricultural development but also provide an effective solution to address climate change.

## 7. Conclusions

As a crucial source of roughage in contemporary animal husbandry, whole-plant corn silage has developed a comprehensive and integrated technological system encompassing variety selection, precision planting, efficient fermentation, and scientific feeding. Recent studies indicate that optimizing variety adaptability, enhancing mechanized harvesting timing, and advancing directional fermentation regulation can significantly improve the dry matter retention rate and nutrient bioavailability of silage. These improvements, in turn, enhance the production performance of ruminants and mitigate the risk of metabolic diseases. Nonetheless, the increasing frequency of extreme weather events, intensified competition for land resources, and the demand for alternatives to antibiotics due to global climate change are compelling the industry to shift towards resource intensification and sustainable development. To align with the goal of achieving carbon neutrality, it is imperative for the whole-plant corn silage sector to develop “climate-smart” production standards, establish a transnational technology-sharing platform, and implement a green subsidy policy framework. These measures are essential to sustain its indispensable role in animal husbandry while maintaining the equilibrium between food security and ecological sustainability.

## Data Availability

No new data were created or analyzed in this study.
